# Outcome data for renal denervation: craving the unattainable?

**DOI:** 10.1038/s41440-024-01667-x

**Published:** 2024-04-15

**Authors:** Lucas Lauder, Felix Mahfoud

**Affiliations:** 1 Klinik für Innere Medizin III—Kardiologie, Angiologie und Internistische Intensivmedizin, Universitätskliniken des Saarlandes, Saarland University, Homburg, Germany; 2 Institute for Medical Engineering and Science, Massachusetts Institute of Technology, Cambridge, MA USA

**Keywords:** Hypertension, Blood pressure, Mortality, Morbidity

From the outset, registries and studies consistently demonstrated the safety of catheter-based renal denervation (RDN); however, there has been considerable controversy regarding its effectiveness in lowering blood pressure. During the last 5 years, the RADIANCE and SPYRAL trial programs have conclusively proven that ultrasound and radiofrequency RDN using the Paradise (ReCor, Palo Alto, CA, USA) and Symplicity Spyral (Medtronic, Santa Rosa, CA, USA) catheter systems lower office and ambulatory BP compared with sham in a broad range of patients with hypertension, including resistant hypertension [1]. These trial programs have led to their approval by the US Food and Drug Administration (FDA) for the treatment of uncontrolled hypertension. In recognition of the evidence coming from second-generation sham-controlled trials, both the 2022 *European Society of Cardiology (ESC)’s Council on Hypertensio*n/*European Association of Percutaneous Cardiovascular Interventions (EAPCI) clinical consensus statement* [1] and the 2023 *European Society of Hypertension (ESH)’s* hypertension guidelines [2] consider RDN a treatment option in patients with resistant hypertension and those with uncontrolled hypertension despite the use of antihypertensive drug combination or if drug treatment elicits serious side effects and poor quality of life (class II recommendation in the ESH guidelines [2]). Additionally, data from three trials conducted in China provide further evidence supporting the BP-lowering efficacy of radiofrequency RDN using the Iberis (CE-marked), Netrod (CE-marked), and SyMap catheter systems. The latter catheter system has a unique approach using electric stimulation to identify hot spots, whose stimulation should increase BP, and which are subsequently ablated, and cold or neutral spots, which should be avoided [3].

In the first part of their review published in this issue of *The Journal*, Haider et al. [4] comprehensively review the statistical methods used by contemporary RDN trials. In the second part, the authors discuss whether cardiovascular (CV) outcome data are needed or if BP reduction is an adequate surrogate outcome. Per definition, surrogates are biomarkers that predict events [5]. The use of surrogates allows for smaller sample sizes than dichotomous variables, shorter follow-up periods, and thereby lower study costs [5]. BP is a surrogate outcome accepted by both clinicians and regulators [6, 7] since BP lowering with first-class agents has robustly shown to reduce CV morbidity and mortality [6]. It is worth noting that there are no CV outcome trials conducted for various antihypertensive treatments, such as exercise, metabolic surgery, mineralocorticoid receptor antagonists, clonidine, moxonidine, and doxazosin. Nevertheless, it is yet to be determined whether the BP lowering following device-based therapies, such as RDN, reduces CV disease events. Only observational studies, which naturally have several limitations, suggested associations between RDN and reduced risk for CV disease events [8, 9]. However, as Haider et al. acknowledge, reducing BP does not necessarily decrease CV events [10]. The authors refer to the ALTITUDE trial, which investigated the impact of aliskiren, a renin inhibitor, compared with placebo in addition to a renin-angiotensin system (RAS) inhibitor in type 2 diabetes [10]. In the trial, BP slightly rose in both treatment groups, but less in the aliskiren than in the placebo group [10]. The trial was stopped after an interim analysis as the primary composite of cardiorenal events did not differ between treatments, but dual RAS blockade resulted in more adverse events, including hyperkalemia, renal dysfunction, and hypotension [10]. Hence, the ALTITUDE trial [10] underscores a general limitation of surrogate markers. In cases where the treatment’s impact on the surrogate marker (e.g., BP-lowering) does not translate into the desired outcome due to known or unknown harmful off-target effects (e.g., hyperkalemia or renal dysfunction) offsetting the overall beneficial impact [7].

As of now, there is no awareness of any harmful off-target effects associated with RDN. Indeed, by reducing sympathetic nervous system activity, RDN could offer advantages over certain medications. It may not only lower BP but also potentially improve other conditions associated with increased sympathetic nervous system activity, including diabetes, atrial fibrillation, metabolic syndrome, and heart failure [11]. Moreover, in contrast to antihypertensive medications, RDN lowers BP continuously over 24 h, regardless of patient’s adherence and independent of pharmacodynamics and -kinetics (“always-on effect”) [1]. Non-adherence is a major contributor to poor BP control rates. Complex medication regimens, including polypharmacy and multiple doses daily, are known to impact adherence [12]. While the majority of patients undergoing RDN may still require antihypertensive medication, the procedure has the potential to decrease the quantity of pills they need to take. Administering additional drugs may introduce complex drug-drug interactions, while it is likely that there is no interaction between RDN and concomitant medications [13]. Reducing night-time BP, which is closely linked with the risk of coronary artery disease and heart failure [14], might be beneficial compared with shorter-acting antihypertensive drugs and is particularly appealing.

Based on recommendations from the GRADE working group [15] the recent ESH guidelines redefined their criteria for level of evidence grading. To assign a level of evidence “A,” well-conducted randomized controlled trials with CV disease outcomes or meta-analysis thereof are required [2]. This is because the primary objective of antihypertensive treatments is to mitigate the risk of cardiovascular outcomes, not solely to address blood pressure. Consequently, an outcome trial must be conducted to fulfill these requirements. As discussed in the 2022 ESC/EAPCI consensus document, outcome trials are challenging to conduct as confounding is likely (changes in adherence, lifestyle modification, etc.), they are expensive, and long-term follow-up would be required [1]. Furthermore, the residual risk observed in recent trials, such as SPRINT and STEP, is very low, necessitating a sample size of up to 20,000 patients [1]. Haider et al. correctly acknowledge that investigating RDN in high-risk patients with a greater absolute risk would allow for a smaller sample size and could be the next “logical step.” On the other hand, a reduction in hypertension-mediated organ damage (e.g., left ventricular hypertrophy, urinary albumin excretion, etc.) could potentially emerge as a substantial outcome in future trials, thereby addressing the evidence gap on whether the BP decrease associated with RDN translates into organ protection.

Asserting that it is impractical to carry out outcome studies may be shortsighted and should not prompt a lowering of our evidence grading standards. The progress in the development of RDN has provided valuable insights, indicating that sham-controlled trials investigating CV interventions are both feasible and essential. Consequently, we should embrace the challenge and strive to overcome the obstacle of conducting CV outcome trials with contemporary trial designs and methods. The narrative doesn’t conclude at this point.
